# Drug Susceptibility of 33 Reference Strains of Slowly Growing Mycobacteria to 19 Antimicrobial Agents

**DOI:** 10.1155/2017/1584658

**Published:** 2017-04-20

**Authors:** Hui Pang, Yi Jiang, Kanglin Wan

**Affiliations:** ^1^Department of Immunology, Changzhi Medical College, Changzhi, Shanxi 046000, China; ^2^State Key Laboratory for Infectious Disease Prevention and Control, National Institute for Communicable Disease Control and Prevention, Chinese Center for Disease Control and Prevention, Beijing 102206, China; ^3^Collaborative Innovation Center for Diagnosis and Treatment of Infectious Diseases, Hangzhou, Zhejiang 310000, China

## Abstract

*Objectives*. Slowly growing mycobacteria (SGM) are prevalent worldwide and cause an extensive spectrum of diseases.* Methods*. In this study, the antimicrobial susceptibility of 33 reference strains of SGM to 19 antimicrobial agents was tested using a modified microdilution method.* Results*. Cefmetazole (32/33) and azithromycin (32/33) exhibited the highest antimicrobial activity, and dapsone (9/33) exhibited the lowest activity against the tested strains. Cefoxitin (30/33), cefoperazone (28/33), and cefepime (28/33) were effective against a high proportion of strains, and macrolides were also highly effective as well as offering the benefit of convenient oral administration to patients. Linezolid (27/33), meropenem (26/33), sulfamethoxazole (26/33), and tigecycline (25/33) showed the highest activity; clofazimine (20/33) and doxycycline (18/33) showed intermediate activity; and rifapentine (13/33), rifabutin (13/33), and minocycline (11/33) showed low antimicrobial activity, closely followed by thioacetazone (10/33) and pasiniazid (10/33), against the tested organisms. According to their susceptibility profiles, the slowly growing species* Mycobacterium avium* and* Mycobacterium simiae* were the least susceptible to the tested drugs, whereas* Mycobacterium intracellulare*,* Mycobacterium asiaticum*,* Mycobacterium scrofulaceum*,* Mycobacterium szulgai*,* Mycobacterium branderi*, and* Mycobacterium holsaticum* were the most susceptible.* Conclusions*. In summary, cephalosporins and macrolides, particularly cefmetazole, azithromycin, clarithromycin, and roxithromycin, showed good antimicrobial activity against the reference strains of SGM.

## 1. Introduction

Slowly growing mycobacteria (SGM) species are ubiquitous organisms that are widely distributed in the environment [1], not only in tap water, soil, dust, and food products but also in domestic and wild animals [2]. SGM form colonies visible to the naked eye in more than 7 days on subculture media [3]. SGM comprise some common species, such as the* Mycobacterium avium* complex (*Mycobacterium avium*,* Mycobacterium intracellulare*, and* Mycobacterium chimaera*),* Mycobacterium kansasii*,* Mycobacterium haemophilum*,* Mycobacterium marinum*, and* Mycobacterium ulcerans*, in addition to some less common pathogens, such as* Mycobacterium scrofulaceum*,* Mycobacterium simiae*,* Mycobacterium malmoense* and* Mycobacterium xenopi*.* Mycobacterium xenopi* is largely distributed in Canada and northern Europe [4]. Slowly growing species were the first nontuberculous mycobacteria (NTM) to be recognized as causing chronic lung disease [4, 5], which may bring about diverse infections from minor sicknesses to serious widespread disorders [6].

At present, standard therapeutic strategies to treat SGM infections are lacking. In this study, 19 new antimicrobial agents were tested against 33 reference SGM pathogens using a modified broth microdilution method with the aim of identifying optimal schemes according to the Clinical Laboratory Standards Institute (CLSI) (USA) [7, 8] and World Health Organization (WHO) [9] guidelines.

## 2. Materials and Methods

### 2.1. Reference Strains

Thirty-three international reference SGM strains were purchased from Deutsche Sammlung von Mikroorganismen und Zellkulturen (DSMZ) and the American Type Culture Collection (ATCC), including* Mycobacterium avium*,* Mycobacterium intracellulare*,* Mycobacterium shimoidei*,* Mycobacterium farcinogenes*, and* Mycobacterium simiae* (Table 1). These strains were cultured at the appropriate temperatures.

### 2.2. Antimicrobial Agents

Nineteen chemicals were purchased from Sigma-Aldrich Company: cefoxitin (FOX), cefoperazone (CFP), cefmetazole (CMZ), cefepime (FEP), rifapentine (RPT), rifabutin (RBT), azithromycin (AZM), clarithromycin (CLR), roxithromycin (ROX), thioacetazone (THI), doxycycline (DOX), minocycline (MIN), tigecycline (TIG), meropenem (MEM), clofazimine (CLO), sulfamethoxazole (SMZ), pasiniazid (PASI), linezolid (LNZ), and dapsone (DAP). All of the antituberculous agents were freshly prepared.

### 2.3. Drug Susceptibility Test

SGM strains were incubated using Difco Middlebrook 7H10 Agar (BD company) with 5% oleic acid-albumin-dextrose-catalase (OADC) [8]. The drug sensitivity tests were performed using a cation-adjusted Mueller-Hinton (CAMH) broth microdilution method, with the addition of 5% OADC, according to the CLSI standard operating procedure [8]. All of the experiments were performed in 96-well microplates and repeated. The minimum inhibitory concentration (MIC) for each antibiotic for each strain was the mean of two experiments. Firstly, the bacterial suspensions were prepared as follows: bacterial inocula were adjusted with normal saline to a density of a 0.5 McFarland standard with an inoculum density of approximately 1 × 10^7^ colony forming units (CFU)/mL; then 50 *μ*L of the bacterial suspension was mixed with 10 mL of CAMH and 5% OADC broth for a 1 : 200 dilution. Secondly, 100 *μ*L of CAMH and 5% OADC medium were added to each well of a 96-well microplate, with the exception of the first well of every row to which 180 *μ*L of medium and a 20 *μ*L drug dilution were added. The solution in the first well was successively diluted into subsequent wells, up to the 11th well. The 12th well in every row was used as a blank control. Finally, 100 *μ*L of the bacterial dilution was added to all of the wells. The ultimate volume in each well was 200 *μ*L. All of the 96-well microplates were sealed in a plastic bag and incubated at 37°C. The concentrations of sulfamethoxazole, dapsone, cefoxitin, cefmetazole, cefoperazone, cefepime, thioacetazone, pasiniazid, minocycline, doxycycline, tigecycline, and meropenem were 0.25–256 *μ*g/mL; the concentrations of clarithromycin, azithromycin, roxithromycin, clofazimine, rifapentine, and rifabutin were 0.03–32 *μ*g/mL; and the concentration of linezolid was 0.06–64 *μ*g/mL. Two negative controls were applied: a no drug control (CAMH + OADC + bacteria) and a no bacteria control (barely CAMH and OADC) [10]. The MIC breakpoints of the drugs exhibiting susceptibility, moderate susceptibility, and resistance were assigned according to the CLSI [7, 8] and WHO [9] guidelines (Table 2).

## 3. Results

The antimicrobial susceptibility profiles of the 33 SGM reference species to 19 antibacterial agents are presented in Table 1. Cephalosporins including cefoxitin (30/33, 90.91%), cefoperazone (28/33, 84.85%), cefmetazole (32/33, 96.97%), and cefepime (28/33, 84.85%) exhibited high activity against the tested strains. Macrolide antibiotics including azithromycin (32/33, 96.97%), clarithromycin (30/33, 90.91%), and roxithromycin (31/33, 93.94%) were also effective against the SGM strains. Linezolid (27/33, 81.82%), meropenem (26/33, 78.79%), and sulfamethoxazole (26/33, 78.79%) showed similar levels of activity against the tested strains, and clofazimine (20/33, 60.61%) inhibited most of the SGM strains. The tetracyclines, doxycycline (18/33, 54.55%), minocycline (11/33, 33.33%), and tigecycline (25/33, 75.76%), exhibited different levels of activity against the SGM standard species, whereas rifapentine (13/33, 39.39%) and rifabutin (13/33, 39.39%) showed weak antimicrobial activity against the SGM, as did thioacetazone (10/33, 30.30%), pasiniazid (10/33, 30.30%), and dapsone (9/33, 27.27%).

**Figure 1:**
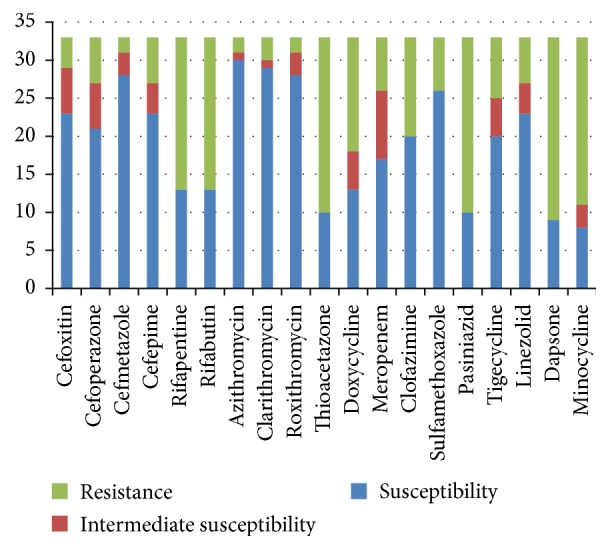
The sensitivity profiles of 33 reference slowly growing mycobacteria to 19 antimicrobial agents.

**Figure 2:**
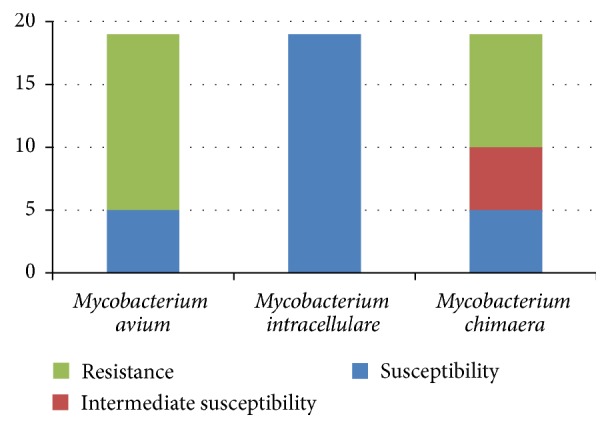
The sensitivity profiles of the* Mycobacterium avium* complex to 19 antimicrobial agents.

The drug susceptibility profiles of the tested organisms revealed that* Mycobacterium avium* and* Mycobacterium simiae* were the least susceptible to the tested drugs, whereas* Mycobacterium intracellulare*,* Mycobacterium asiaticum*,* Mycobacterium scrofulaceum*,* Mycobacterium szulgai*,* Mycobacterium branderi*, and* Mycobacterium holsaticum* were the most susceptible (Table 3 and Figure 1). Among the* Mycobacterium avium* complex,* Mycobacterium avium* was the most resistant to the tested drugs, whereas* Mycobacterium intracellulare* was the most susceptible (Figure 2). Azithromycin was identified as the most effective antimicrobial agent against SGM species among the drugs tested, and dapsone was the least effective.

## 4. Discussion

In this study, 19 antimicrobial susceptibility tests were performed against 33 SGM organisms by a Microplate Alamar Blue Assay. The current first-line drugs for the treatment of nontuberculous mycobacteria are capreomycin, clarithromycin, and rifampin. And the current second-line drugs for the treatment of nontuberculous mycobacteria are moxifloxacin, linezolid, amikacin, ciprofloxacin, ethambutol, isoniazid, rifabutin, streptomycin, and trimethoprim-sulfamethoxazole [7]. Our findings indicated that cephalosporins and macrolides, particularly cefmetazole, azithromycin, clarithromycin, and roxithromycin, showed effective antimicrobial activity against the tested strains.

In recent studies [4, 11–15], cefoxitin and meropenem have been reported to show some activity against* Mycobacterium abscessus*,* Mycobacterium chelonae*, and* Mycobacterium fortuitum*, whereas* Mycobacterium kansasii* has been shown to be susceptible to clarithromycin and linezolid. Macrolides were active against isolates of* Mycobacterium avium* [12, 16, 17], and tigecycline has been demonstrated to exhibit high level antimicrobial activity against RGM in vitro [18]. In other studies,* Mycobacterium kansasii* was reported to be the most susceptible NTM species in vitro [19], and* Mycobacterium simiae* was found to be resistant to clarithromycin, doxycycline, and sulfamethoxazole [20, 21]. However, few studies have tested the activity of cefoperazone, cefmetazole, and cefepime against SGM. In our study, cephalosporins were found to be effective antimicrobial agents and cefmetazole in particular was identified as a good candidate for the treatment of SGM infections. In previous research [15, 22], clarithromycin has been widely used as an antimicrobial agent to SGM, whereas azithromycin and roxithromycin have rarely been tested. Among the tetracyclines, tigecycline was found to be the most effective against SGM. Previous studies have reported that* Mycobacterium kansasii* was 100% resistant to doxycycline, and* Mycobacterium simiae* isolates were 100% resistant to clarithromycin, doxycycline, and sulfamethoxazole.

*Mycobacterium avium* and* Mycobacterium intracellulare* are important members of the SGM. Macrolides and sulfamethoxazole are recognized as useful drugs against* Mycobacterium avium* and* Mycobacterium intracellulare*, but rifapentine is ineffective against* Mycobacterium avium*.* Mycobacterium chimaera*, a recently described species distinct from* Mycobacterium intracellulare*, is regarded as less virulent than* Mycobacterium intracellulare* [23, 24], but neither rifapentine nor rifabutin was effective against* Mycobacterium chimaera*.

*Mycobacterium simiae* was highly resistant to the tested drugs. It was first isolated from monkeys in 1965 and is now most frequently isolated from human respiratory specimens [25, 26], predominantly being reported in the southwest of the United States and Middle Eastern countries, including Israel and Iran [27].

## 5. Conclusions

Our findings present the drug susceptibility profiles of representative SGM species to a range of antimicrobial agents and provide insight into potentially effective therapeutic strategies. In the future, susceptibility testing of clinical isolates may help to tailor therapeutic strategies to individual patients. Combination therapy should also be explored as a means to increase the efficacy of drug treatment against SGM pathogens. Furthermore, the synergistic activity of some drugs will be analyzed, and drug susceptibility in vivo response must be performed in our recent research.

## Figures and Tables

**(a):** 

Sp. (international code)	FOX	CFP	CMZ	FEP	RPT	RBT	AZM	CLR	ROX	THI
*Mycobacterium avium* (DSM44133)	>256	128	>256	>256	4	**0.25**	**4**	**0.5**	**1**	>256
*Mycobacterium intracellulare* (ATCC13950)	**0.5**	**8**	**1**	**4**	**<0.03**	**<0.03**	**<0.03**	**0.13**	**<0.03**	**<0.25**
*Mycobacterium shimoidei *(ATCC27962)	**8**	**16**	**4**	>256	2	**0.06**	**0.13**	**<0.03**	**0.06**	>256
*Mycobacterium farcinogenes* (ATCC35753)	**4**	**16**	**2**	**<0.25**	**<0.03**	**<0.03**	**0.13**	**<0.03**	**<0.03**	**2**
*Mycobacterium simiae* (ATCC25275)	>256	256	256	128	16	4	**16**	**1**	**4**	256
*Mycobacterium asiaticum* (ATCC25276)	**2**	**<0.25**	**<0.25**	**1**	4	**<0.03**	**0.5**	**<0.03**	**0.13**	**1**
*Mycobacterium scrofulaceum* (ATCC19981)	**64**	**64**	**32**	**32**	**<0.03**	**<0.03**	**2**	**0.06**	**0.13**	**<0.25**
*Mycobacterium szulgai* (ATCC35799)	**4**	**2**	**2**	**1**	**<0.03**	**<0.03**	**0.13**	**<0.03**	**<0.03**	256
*Mycobacterium africanum* (ATCC35711)	**64**	**64**	**8**	256	16	32	32	32	32	>256
*Mycobacterium alvei* (DSM44176)	**16**	**64**	**0.5**	**<0.25**	1	**0.13**	**0.06**	**0.06**	**<0.03**	256
*Mycobacterium branderi* (ATCC51788)	**4**	**16**	**1**	**16**	32	**0.25**	**0.25**	**<0.03**	**<0.03**	>256
*Mycobacterium celatum* (ATCC44243)	128	128	**64**	**64**	32	**0.5**	**1**	**0.06**	**0.25**	128
*Mycobacterium chimaera* (DSM44623)	**64**	**32**	**16**	**64**	16	16	**8**	**0.5**	**0.5**	64
*Mycobacterium cosmeticum* (DSM44829)	**64**	>256	**8**	256	>32	32	**2**	**0.25**	**1**	>256
*Mycobacterium duvalii* (ATCC43910)	**<0.25**	**16**	**1**	**0.5**	**0.06**	**<0.03**	**0.5**	**0.06**	**0.13**	8
*Mycobacterium elephantis* (DSM44368)	**8**	256	**2**	**4**	8	2	**8**	**1**	**4**	32
*Mycobacterium hassiacum* (DSM44199)	128	>256	**16**	256	32	**0.5**	**<0.03**	**<0.03**	**<0.03**	>256
*Mycobacterium hiberniae* (DSM44241)	**1**	**2**	**<0.25**	**16**	**0.25**	**<0.03**	**2**	**0.06**	**0.13**	64
*Mycobacterium holsaticum* (DSM44478)	**1**	**32**	**<0.25**	**0.5**	4	1	**<0.03**	**<0.03**	**<0.03**	256
*Mycobacterium houstonense* (DSM44676)	**32**	**32**	**64**	**32**	64	>32	32	**8**	**2**	64
*Mycobacterium kubicae* (DSM44627)	**64**	**16**	**0.5**	**16**	64	>32	**2**	**<0.25**	**16**	256
*Mycobacterium lentiflavum* (DSM44418)	**2**	**4**	**4**	**0.13**	**0.5**	>32	**8**	**2**	32	>256
*Mycobacterium mageritense* (DSM44476)	**8**	**1**	**0.5**	**8**	32	>32	**8**	**4**	***16***	64
*Mycobacterium nonchromogenicum* (DSM44164)	**2**	**<0.25**	**4**	**<0.25**	1	>32	**8**	**2**	**<0.03**	**2**
*Mycobacterium palustre *(DSM44572)	**<0.25**	**<0.25**	**0.5**	**<0.25**	**0.06**	>32	**<0.03**	**0.5**	**0.06**	**4**
*Mycobacterium parascrofulaceum* (DSM44648)	**<0.25**	**<0.25**	**4**	**0.25**	**0.06**	8	**1**	**16**	**<0.03**	64
*Mycobacterium senuense* (DSM44999)	**0.5**	**<0.25**	**2**	**<0.25**	**<0.03**	32	**1**	**<0.25**	**0.06**	**2**
*Mycobacterium seoulense* (DSM44998)	**<0.25**	**<0.25**	**4**	**<0.25**	**0.5**	>32	**2**	32	**<0.03**	128
*Mycobacterium thermoresistibile* (DSM44167)	**8**	**0.25**	**4**	**2**	2	>32	**2**	**1**	**1**	**2**
*Mycobacterium triplex* (DSM44626)	**0.25**	**0.5**	**<0.25**	**1**	4	>32	**1**	**<0.25**	**0.13**	**0.5**
*Mycobacterium vanbaalenii* (DSM7251)	**8**	**0.25**	**0.5**	**1**	**0.06**	>32	**<0.25**	64	**1**	256
*Mycobacterium murale* (DSM44340)	**0.5**	**<0.25**	**0.25**	**0.25**	**0.25**	>32	**<0.25**	**8**	**<0.03**	**2**
*Mycobacterium gordonae (*ATCC14470)	**4**	**0.5**	**16**	**0.5**	1	>32	**8**	**8**	**16**	128

**(b):** 

Sp. (international code)	DOX	MIN	TIG	MEM	CLO	SMZ	PASI	LNZ	DAP
*Mycobacterium avium* (DSM44133)	>256	64	32	>256	16	**32**	32	32	>256
*Mycobacterium intracellulare* (ATCC13950)	**<0.25**	**<0.25**	**0.5**	**0.5**	**0.13**	**<0.25**	**<0.25**	**<0.06**	**<0.25**
*Mycobacterium shimoidei *(ATCC27962)	256	64	**1**	>256	**0.13**	**4**	**0.5**	**0.5**	64
*Mycobacterium farcinogenes* (ATCC35753)	**0.25**	8	**<0.03**	**<0.25**	**<0.03**	**1**	16	**0.25**	4
*Mycobacterium simiae* (ATCC25275)	>256	16	16	>256	**<0.03**	**16**	16	32	16
*Mycobacterium asiaticum* (ATCC25276)	**<0.25**	**0.5**	**<0.03**	**<0.25**	**<0.03**	**<0.25**	2	**0.13**	**<0.25**
*Mycobacterium scrofulaceum* (ATCC19981)	**0.25**	**2**	**0.5**	32	**<0.03**	**16**	**1**	**1**	**2**
*Mycobacterium szulgai* (ATCC35799)	**<0.25**	**<0.25**	**0.13**	**0.5**	**<0.03**	**<0.25**	**1**	**0.13**	**<0.25**
*Mycobacterium africanum* (ATCC35711)	**4**	8	**0.25**	**4**	32	256	>256	**16**	4
*Mycobacterium alvei* (DSM44176)	**<0.25**	32	**<0.03**	**<0.25**	**0.5**	**4**	8	**16**	64
*Mycobacterium branderi* (ATCC51788)	**<0.25**	**0.5**	**0.06**	**0.5**	**<0.03**	**0.5**	**1**	**<0.06**	**<0.25**
*Mycobacterium celatum* (ATCC44243)	**2**	**0.5**	**4**	64	**<0.03**	**2**	**0.5**	**16**	**<0.25**
*Mycobacterium chimaera* (DSM44623)	8	32	***4***	32	**0.5**	64	>256	**16**	64
*Mycobacterium cosmeticum* (DSM44829)	**2**	128	**1**	**8**	32	256	>256	32	256
*Mycobacterium duvalii* (ATCC43910)	**<0.25**	16	**<0.03**	**2**	1	**4**	**0.5**	**0.25**	8
*Mycobacterium elephantis* (DSM44368)	**<0.25**	**0.5**	**<0.03**	**8**	**0.13**	**<0.25**	**0.5**	**0.5**	**0.5**
*Mycobacterium hassiacum* (DSM44199)	**<0.25**	8	**<0.03**	256	**0.13**	**8**	8	**4**	16
*Mycobacterium hiberniae* (DSM44241)	**1**	8	**0.13**	**4**	**<0.03**	**1**	8	**1**	4
*Mycobacterium holsaticum* (DSM44478)	**<0.25**	**1**	**0.25**	**16**	**0.13**	**<0.25**	**1**	**0.5**	**0.5**
*Mycobacterium houstonense* (DSM44676)	64	64	16	**16**	32	256	64	32	256
*Mycobacterium kubicae* (DSM44627)	>256	16	***2***	**2**	32	>256	16	32	>256
*Mycobacterium lentiflavum* (DSM44418)	>256	128	**4**	**4**	4	128	128	**4**	128
*Mycobacterium mageritense* (DSM44476)	>256	128	8	**8**	32	**8**	128	32	8
*Mycobacterium nonchromogenicum* (DSM44164)	128	128	16	**16**	2	**4**	128	**2**	4
*Mycobacterium palustre *(DSM44572)	**2**	32	**0.06**	**<0.25**	**0.25**	**8**	32	**0.25**	8
*Mycobacterium parascrofulaceum*(DSM44648)	64	32	**0.03**	**<0.25**	2	**32**	32	**2**	32
*Mycobacterium senuense* (DSM44999)	**1**	**4**	**1**	**1**	**0.5**	**4**	4	**0.5**	4
*Mycobacterium seoulense* (DSM44998)	**2**	128	8	**8**	4	**8**	128	**4**	8
*Mycobacterium thermoresistibile* (DSM44167)	256	256	***2***	**2**	1	**8**	256	**1**	8
*Mycobacterium triplex* (DSM44626)	256	**0.5**	**0.06**	**<0.25**	**0.13**	**0.5**	**0.5**	**0.13**	**0.5**
*Mycobacterium vanbaalenii* (DSM7251)	8	**4**	8	**8**	2	**4**	4	**2**	4
*Mycobacterium murale* (DSM44340)	256	16	**0.06**	**<0.25**	**0.5**	**8**	16	**0.5**	8
*Mycobacterium gordonae (*ATCC14470)	>256	16	16	**16**	**0.5**	256	16	**0.5**	256

*Note 1*. FOX: cefoxitin; CFP: cefoperazone; CMZ: cefmetazole; FEP: cefepime; RPT: rifapentine; RBT: rifabutin; AZM: azithromycin; CLR: clarithromycin; ROX: roxithromycin; THI: thioacetazone; DOX: doxycycline; MIN: minocycline; TIG: tigecycline; MEM: meropenem; CLO: clofazimine; SMZ: sulfamethoxazole; PASI: pasiniazid; LNZ: linezolid; DAP: dapsone.

*Note 2*. Bold numbers indicate drug susceptibility. Numbers in bold and cursive indicate intermediate drug susceptibility.

**Table 2:** The MIC (*μ*g/mL) breakpoints of 19 antibacterial agents.

	Susceptibility	Intermediate susceptibility	Resistance
Cefoxitin	≤16	32–64	≥128
Cefoperazone	≤16	32–64	≥128
Cefmetazole	≤16	32–64	≥128
Cefepime	≤16	32–64	≥128
Rifapentine	—	—	>1
Rifabutin	—	—	>2
Azithromycin	≤8	16	≥32
Clarithromycin	≤8	16	≥32
Roxithromycin	≤8	16	≥32
Thioacetazone	—	—	≥8
Doxycycline	≤1	2–4	≥8
Meropenem	≤4	8–16	≥32
Clofazimine	—	—	≥1
Sulfamethoxazole	≤38	—	≥76
Pasiniazid	—	—	≥2
Minocycline	≤1	2–4	≥8
Linezolid	≤8	16	≥32
Dapsone	—	—	≥4
Tigecycline	≤1	2–4	≥8

**Table 3:** Susceptibility of 33 international standard slowly growing mycobacterial strains to 19 antibacterial agents.

Sp. (international code)	FOX	CFP	CMZ	FEP	RPT	RBT	AZM	CLR	ROX	THI	DOX	MIN	TIG	MEM	CLO	SMZ	PASI	LNZ	DAP	Susceptibility rate (%)
*Mycobacterium avium* (DSM44133)	−	−	−	−	−	+	+	+	+	−	−	−	−	−	−	+	−	−	−	26.32
*Mycobacterium intracellulare* (ATCC13950)	+	+	+	+	+	+	+	+	+	+	+	+	−	+	+	+	+	+	+	94.74
*Mycobacterium shimoidei *(ATCC27962)	+	+	+	−	−	+	+	+	+	−	−	−	+	−	+	+	+	+	−	63.16
*Mycobacterium farcinogenes* (ATCC35753)	+	+	+	+	+	+	+	+	+	+	+	−	+	+	+	+	−	+	−	84.21
*Mycobacterium simiae* (ATCC25275)	−	−	−	−	−	−	+	+	+	−	−	−	−	−	+	+	−	−	−	26.32
*Mycobacterium asiaticum* (ATCC25276)	+	+	+	+	+	+	+	+	+	+	+	+	+	+	+	+	−	+	+	94.74
*Mycobacterium scrofulaceum* (ATCC19981)	+	+	+	+	+	+	+	+	+	+	+	+	+	−	+	+	+	+	+	94.74
*Mycobacterium szulgai* (ATCC35799)	+	+	+	+	+	+	+	+	+	−	+	+	+	+	+	+	+	+	+	94.74
*Mycobacterium africanum* (ATCC35711)	+	+	+	−	+	+	+	−	−	−	+	−	+	+	−	−	−	+	−	52.63
*Mycobacterium alvei* (DSM44176)	+	+	+	+	+	+	+	+	+	−	+	−	+	+	+	+	−	+	−	78.95
*Mycobacterium branderi* (ATCC51788)	+	+	+	+	+	+	+	+	+	−	+	+	+	+	+	+	+	+	+	94.74
*Mycobacterium celatum* (ATCC44243)	−	−	+	+	+	+	+	+	+	−	+	+	+	−	+	+	+	+	+	78.95
*Mycobacterium chimaera* (DSM44623)	+	+	+	+	+	+	+	+	+	−	−	−	+	−	+	−	−	+	−	63.16
*Mycobacterium cosmeticum* (DSM44829)	+	−	+	−	+	+	+	+	+	−	+	−	+	+	−	−	−	−	−	52.63
*Mycobacterium duvalii* (ATCC43910)	+	+	+	+	+	+	+	+	+	−	+	−	+	+	−	+	+	+	−	78.95
*Mycobacterium elephantis* (DSM44368)	+	−	+	+	+	+	+	+	+	−	+	+	+	+	+	+	+	+	+	89.47
*Mycobacterium hassiacum* (DSM44199)	−	−	+	−	+	+	+	+	+	−	+	−	+	−	+	+	−	+	−	57.89
*Mycobacterium hiberniae* (DSM44241)	+	+	+	+	+	+	+	+	+	−	+	−	+	+	+	+	−	+	−	78.95
*Mycobacterium holsaticum* (DSM44478)	+	+	+	+	+	+	+	+	+	−	+	+	+	+	+	+	+	+	+	94.74
*Mycobacterium houstonense* (DSM44676)	+	+	+	+	+	−	+	+	+	−	−	−	−	+	−	−	−	−	−	47.37
*Mycobacterium kubicae* (DSM44627)	+	+	+	+	+	−	+	+	+	−	−	−	+	+	−	−	−	−	−	52.63
*Mycobacterium lentiflavum* (DSM44418)	+	+	+	+	+	−	+	+	−	−	−	−	+	+	−	−	−	+	−	52.63
*Mycobacterium mageritense* (DSM44476)	+	+	+	+	+	−	+	+	+	−	−	−	−	+	−	+	−	−	−	52.63
*Mycobacterium nonchromogenicum* (DSM44164)	+	+	+	+	+	+	+	+	+	+	−	−	−	+	−	+	−	+	−	68.42
*Mycobacterium palustre *(DSM44572)	+	+	+	+	+	+	+	+	+	+	+	−	+	+	+	+	−	+	−	84.21
*Mycobacterium parascrofulaceum* (DSM44648)	+	+	+	+	+	+	+	+	+	−	−	−	+	+	−	+	−	+	−	68.42
*Mycobacterium senuense* (DSM44999)	+	+	+	+	+	+	+	+	+	+	+	+	+	+	+	+	−	+	−	89.47
*Mycobacterium seoulense* (DSM44998)	+	+	+	+	+	−	+	−	+	−	+	−	−	+	−	+	−	+	−	57.89
*Mycobacterium thermoresistibile* (DSM44167)	+	+	+	+	+	−	+	+	+	+	−	−	+	+	−	+	−	+	−	68.42
*Mycobacterium triplex* (DSM44626)	+	+	+	+	+	−	+	+	+	+	−	+	+	+	+	+	+	+	+	89.47
*Mycobacterium vanbaalenii* (DSM7251)	+	+	+	+	+	+	+	−	+	−	−	+	−	+	−	+	−	+	−	63.16
*Mycobacterium murale* (DSM44340)	+	+	+	+	+	−	+	+	+	+	−	−	+	+	+	+	−	+	−	73.68
*Mycobacterium gordonae (*ATCC14470)	+	+	+	+	+	−	+	+	+	−	−	−	−	+	+	−	−	+	−	57.89

*Note*. “+” indicates sensitivity; “−” indicates resistance.

## References

[1] Aboagye S. Y., Danso E., Ampah K. A. (2016). Isolation of nontuberculous mycobacteria from the environment of Ghanian communities where buruli ulcer is endemic. *Applied and Environmental Microbiology*.

[2] Azadi D., Shojaei H., Pourchangiz M., Dibaj R., Davarpanah M., Naser A. D. (2016). Species diversity and molecular characterization of nontuberculous mycobacteria in hospital water system of a developing country, Iran. *Microbial Pathogenesis*.

[3] Tsukatani T., Suenaga H., Shiga M. (2015). Rapid susceptibility testing for slowly growing nontuberculous mycobacteria using a colorimetric microbial viability assay based on the reduction of water-soluble tetrazolium WST-1. *European Journal of Clinical Microbiology and Infectious Diseases*.

[4] Philley J. V., Griffith D. E. (2015). Treatment of slowly growing mycobacteria. *Clinics in Chest Medicine*.

[5] Ferro B. E., van Ingen J., Wattenberg M., van Soolingen D., Mouton J. W. (2015). Time-kill kinetics of slowly growing mycobacteria common in pulmonary disease. *Journal of Antimicrobial Chemotherapy*.

[6] Griffith D. E. (2010). Nontuberculous mycobacterial lung disease. *Current Opinion in Infectious Diseases*.

[7] Clinical and Laboratory Standards Institute (2011). *Susceptibility Testing of Mycobacteria, Nocardiae, and Other Aerobic Actinomycetes; Approved Standard—Second Edition*.

[8] Clinical and Laboratory Standards Institute (2015). *Performance Standards for Antimicrobial Susceptibility Testing; Twenty First Informational Supplement*.

[9] World Health Organization (2008). *Policy Guidance on Drug-Susceptibility Testing (DST) of Second-Line Antituberculosis Drugs*.

[10] Foongladda S., Pholwat S., Eampokalap B., Kiratisin P., Sutthent R. (2009). Multi-probe real-time PCR identification of common mycobacterium species in blood culture broth. *Journal of Molecular Diagnostics*.

[11] Pang H., Li G., Zhao X., Liu H., Wan K., Yu P. (2015). Drug susceptibility testing of 31 antimicrobial agents on rapidly growing mycobacteria isolates from China. *BioMed Research International*.

[12] Heidarieh P., Mirsaeidi M., Hashemzadeh M. (2016). In vitro antimicrobial susceptibility of nontuberculous mycobacteria in iran. *Microbial Drug Resistance*.

[13] Pang H., Li G., Wan L. (2015). In vitro drug susceptibility of 40 international reference rapidly growing mycobacteria to 20 antimicrobial agents. *International Journal of Clinical and Experimental Medicine*.

[14] Jankovic M., Zmak L., Krajinovic V. (2011). A fatal Mycobacterium chelonae infection in an immunosuppressed patient with systemic lupus erythematosus and concomitant Fahr's syndrome. *Journal of Infection and Chemotherapy*.

[15] Gitti Z., Mantadakis E., Maraki S., Samonis G. (2011). Clinical significance and antibiotic susceptibilities of nontuberculous mycobacteria from patients in Crete, Greece. *Future Microbiology*.

[16] Shah M., Relhan N., Kuriyan A. E. (2016). Endophthalmitis caused by nontuberculous mycobacterium: clinical features, antimicrobial susceptibilities, and treatment outcomes. *American Journal of Ophthalmology*.

[17] Bax H. I., Bakker-Woudenberg I. A. J. M., Kate M. T. T., Verbon A., De Steenwinkelb J. E. M. (2016). Tigecycline potentiates clarithromycin activity against mycobacterium avium in vitro. *Antimicrobial Agents and Chemotherapy*.

[18] Tang S. S., Lye D. C., Jureen R., Sng L.-H., Hsu L. Y. (2015). Rapidly growing mycobacteria in Singapore, 2006–2011. *Clinical Microbiology and Infection*.

[19] Hombach M., Somoskövi A., Hömke R., Ritter C., Böttger E. C. (2013). Drug susceptibility distributions in slowly growing non-tuberculous mycobacteria using MGIT 960 TB eXiST. *International Journal of Medical Microbiology*.

[20] Cowman S., Burns K., Benson S., Wilson R., Loebinger M. R. (2016). The antimicrobial susceptibility of non-tuberculous mycobacteria. *Journal of Infection*.

[21] García-Martos P., García-Agudo L., González-Moya E., Galán F., Rodríguez-Iglesias M. (2015). Infections due to Mycobacterium simiae. *Enfermedades Infecciosas y Microbiologia Clinica*.

[22] Ahmed I., Jabeen K., Hasan R. (2013). Identification of non-tuberculous mycobacteria isolated from clinical specimens at a tertiary care hospital: a cross-sectional study. *BMC Infectious Diseases*.

[23] Moon S. M., Kim S. Y., Jhun B. W. (2016). Clinical characteristics and treatment outcomes of pulmonary disease caused by Mycobacterium chimaera. *Diagnostic Microbiology and Infectious Disease*.

[24] Wallace R. J., Iakhiaeva E., Williams M. D. (2013). Absence of Mycobacterium intracellulare and presence of Mycobacterium chimaera in household water and biofilm samples of patients in the United States with Mycobacterium avium complex respiratory disease. *Journal of Clinical Microbiology*.

[25] Jeong S. H., Kim S.-Y., Lee H. (2015). Nontuberculous mycobacterial lung disease caused by mycobacterium simiae: the first reported case in South Korea. *Tuberculosis and Respiratory Diseases*.

[26] Griffith D. E., Aksamit T., Brown-Elliott B. A. (2007). An official ATS/IDSA statement: diagnosis, treatment, and prevention of nontuberculous mycobacterial diseases. *American Journal of Respiratory and Critical Care Medicine*.

[27] Velayati A. A., Farnia P., Mozafari M. (2014). Molecular epidemiology of nontuberculous mycobacteria isolates from clinical and environmental sources of a metropolitan city. *PLoS ONE*.

